# CD44 Predisposes the Epileptogenesis of Cortical Tubers by Regulating CASK and Acts as a Potential Biomarker to Identify Epileptogenic Tuber in Tuberous Sclerosis Complex

**DOI:** 10.1002/cns.71262

**Published:** 2026-11-09

**Authors:** Zhongke Wang, Kaixuan Huang, Xingye Liu, Yang Li, Shujing Li, Jie Li, Hu Li, Jingze Wang, Zeng He, Limeng Sun, Yali Cheng, Ruotong Ruan, Zhonghong Liu, Shengqing Lv, Chunqing Zhang, Hui Yang, Xiaolin Yang, Shiyong Liu

**Affiliations:** ^1^ Epilepsy Research Center of PLA, Department of Neurosurgery Xinqiao Hospital, Army Medical University Chongqing P.R. China; ^2^ Department of Neurosurgery Armed Police Hospital of Chongqing Chongqing P.R. China; ^3^ Department of Virology, State Key Laboratory of Pathogen and Biosecurity Beijing Institute of Microbiology and Epidemiology, AMMS Beijing P.R. China; ^4^ Chongqing Institute for Brain and Intelligence, Guangyang Bay Laboratory Chongqing P.R. China

**Keywords:** biomarker, CD44, cortical tubers, epileptogenesis, tuberous sclerosis complex

## Abstract

**Background:**

Tuberous sclerosis complex (TSC) is characterized by developmental delay and intractable epilepsy, with multiple cortical tubers in the brain. However, the mechanism underlying epileptogenesis within these tubers remains unclear.

**Methods:**

RNA sequencing (RNA‐seq) analysis of resected human cortical tubers identified differential CD44 expression as a potential contributor to TSC epilepsy. By analyzing clinical characteristics, resected brain tissues, ^18^F‐fludeoxyglucose PET images, cultured cortical neurons, and neocortical seizure animal models, the effect of CD44 on the epileptogenesis of cortical tubers was investigated.

**Results:**

Increased CD44 expression was found in human Epi tubers compared with non‐Epi tubers and closely correlated with the clinical characteristics of Epi tubers. CD44 activation increased neocortical seizure susceptibility and aggravated cortical pyramidal neuronal excitatory/inhibitory (E/I) imbalance. CASK was selected as the downstream target of CD44 using RNA‐seq. Anti‐CD44 monoclonal antibodies rescued the E/I imbalance in resected Epi tubers, and CD44 activation rescued the decreased firing frequency and increased action potential threshold induced by CASK inhibition. The CD44–CASK pathway exhibited diagnostic potential for identifying Epi tubers and predicting surgical outcomes via ROC analysis.

**Conclusion:**

CD44 activation predisposes cortical tubers to epileptogenesis, and could be a potential biomarker for identifying Epi tubers and predicting surgical outcomes in TSC patients.

## Graphical abstract

CD44 activation predisposes cortical tubers to epileptogenesis in patients with TSC. CD44 regulates the cortical neuronal excitability and epileptogenesis of cortical tubers through the CASK signaling pathway in patients with TSC. CD44 could be a potential biomarker in identifying epileptic tubers and predicting surgical outcomes in patients with TSC.

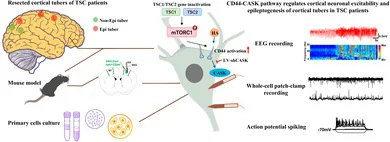

**Abbreviations**
ACSF: artificial cerebrospinal fluid
AP: action potential
CASK: calmodulin‐dependent serine protein kinase
CD44: Cluster of Differentiation 44
co‐IP: co‐immunoprecipitation
CT: computed tomography
DEGs: differential expression genes
DRE: drug‐resistant epilepsy
E/I: excitatory/inhibitory
ECM: extracellular matrix
EEG: electroencephalogram
Epi tubers: epileptogenic tubers
EPSC: excitatory postsynaptic current
FC: fold‐change
FDG: ^18^F‐fludeoxyglucose
FDR: false discovery rate
GO: Gene ontology
GS: generalized seizures
IF: immunofluorescence
IHC: immunohistochemistry
IPSC: inhibitory postsynaptic current
KA: kainic acid
KEGG: Kyoto Encyclopedia of Genes and Genomes
M1: primary motor cortex
MOD: mean optical density
MRI: magnetic resonance imaging
NMDA: N‐methyl‐D‐aspartate
non‐Epi tubers: non‐epileptogenic tubers
OD: optical density
PET: positron emission tomography
PPI: protein–protein interaction
RNA‐seq: RNA sequencing
ROC: receiver operating characteristic
ROI: regions of interest
RT‐qPCR: reverse transcription‐quantitative PCR
SEEG: stereoelectroencephalogram
SUV: standardized uptake value
TSC: tuberous sclerosis complex

## 1 Introduction

Tuberous sclerosis complex (TSC), a rare autosomal dominant genetic disease with an incidence of 1 in 6000–22,000 individuals, presents with diverse clinical features including developmental delay, intellectual disability, behavioral abnormalities and seizures [1, 2, 3, 4]. TSC is a highly complex and variable disease; however, fewer than half of patients with TSC receive adequate care in adherence to TSC patient management guidelines [5]. Epilepsy is the most common neurological manifestation in TSC, with ≥ 50% of patients exhibiting drug‐resistant epilepsy (DRE) for which surgery is the most effective treatment [2, 3, 6, 7]. The 2021 international TSC diagnostic criteria replaced “cortical dysplasias” with “multiple cortical tubers and/or radial migration lines” [2], indicating that multiple cortical tubers are crucial in TSC‐related epilepsy. Cortical tubers can be divided into epileptogenic (Epi) and non‐Epi tubers based on whether or not epileptic discharges can be recorded within them, and Epi tubers were regarded as the origin foci of seizures [6, 7]. Therefore, accurate identification of Epi tubers is critical for precise surgical intervention in TSC.

Comprehensive preoperative assessments, including genetic tests, CT, MRI, PET, and video‐electroencephalogram (EEG) recordings, are vital for identifying Epi tubers from multiple cortical tubers [8, 9, 10, 11], and precise localization of Epi tubers may help determine the surgical approach, outcomes and prognosis of TSC‐related DRE [12, 13, 14]. Epi tubers present typical pathological characteristics, such as dysmorphic neurons and bright eosinophilic giant cells [2]. These malformed neurons exhibit excitatory/inhibitory (E/I) imbalance, which is accompanied by upregulated glutamate transporters, N‐methyl‐D‐aspartate (NMDA) receptors and excitatory postsynaptic current (EPSC) frequency, as well as downregulated GABAergic transporters, GABA receptors and inhibitory postsynaptic current (IPSC) frequency [8, 15, 16, 17]. The increase in the E/I ratio contributes to cortical hyperexcitability, excess spiking and epileptogenesis in Epi tubers [2, 18]. Current clinical methods for Epi tubers identification are diverse, comprehensive and effective [14, 19], but molecular biomarkers regulating the tubers epileptogenesis are lacking.

The extracellular matrix (ECM), comprising 20% of the brain volume, is present from the onset of brain development and regulates neural progenitor cell proliferation, neuronal differentiation and migration, and cortical layer formation in cortical neurogenesis [20, 21]. Notably, ECM alterations play a key role during the development of a hyperexcitable network, and various proteins mediating and regulating cell‐ECM interactions exhibit altered expression patterns following an Epi brain insult, including upregulated CD44 expression [22, 23]. CD44 is an important cell adhesion molecule that interacts with the ECM. It is widely expressed in cortical neurons and is involved in axonal pathfinding, cortical structure and brain plasticity [24, 25, 26]. CD44 shows strong reactivity in both pilocarpine‐ and kainic acid (KA)‐induced seizures, as well as various epilepsy‐related neurological disorders, and regulates the progression of epileptogenesis in mouse hippocampus [24, 27, 28]. In addition, transcriptome analysis in peripheral blood revealed that upregulated CD44 expression was associated with the pathogenesis of epilepsy [29]. However, the role of the ECM and CD44 in the epileptogenesis of cortical tubers and the transformation of Epi tubers from multiple cortical tubers remains largely unknown.

The present study explored the role of CD44 in the epileptogenesis and localization of Epi tubers in TSC. The expression of CD44 in resected human Epi and non‐Epi tubers, as well as the association between the clinical characteristics of Epi tubers with CD44, and the effects of CD44 in neuronal E/I imbalance and hyperexcitability were investigated. In addition, RNA sequencing (RNA‐seq) was performed to explore the target regulated by CD44 on the epileptogenesis of cortical tubers. Finally, the potential value of CD44 in the identification of Epi tubers from multiple cortical tubers was evaluated.

## 2 Material and Methods

### 2.1 Human Subjects

Patients with TSC who underwent epilepsy surgery at the Comprehensive Epilepsy Center of Xinqiao Hospital between June 2017 to January 2023 were retrospectively selected (Figure S1), and the inclusion and exclusion criteria were based on our previous study [30]. Patients received comprehensive preoperative examination, including genetic tests, MRI, ^18^F‐fludeoxyglucose (FDG) PET/CT, scalp EEG, and stereoelectroencephalogram (SEEG) recordings. Informed consent for resected brain tissue use was obtained from all subjects. Resected Epi and non‐Epi tubers were collected. Non‐Epi tubers were anatomically adjacent to Epi tubers within the same operative field, typically located in non‐functional cortical regions and lacked epileptic discharges [7, 31]. All specimens were histopathologically confirmed by two neuropathologists and processed for immunohistochemistry (IHC), immunofluorescence (IF), RT‐qPCR, western blotting, and RNA‐seq.

### 2.2 Animals

Male C57BL/6 mice (8 weeks old, 25–30 g) were housed 5 per cage under standard conditions (12‐h light/dark cycle, 23°C–26°C, 50%–60% humidity, ad libitum food/water) and individually housed after surgery. Male mice were used to eliminate confounding effects of ovarian hormone fluctuations on seizure phenotypes, which would introduce substantial variability in seizure threshold, severity, and survival rate and obscure the specific molecular effects of CD44 we aimed to elucidate [32, 33, 34]. And previous studies investigating fundamental epileptogenesis mechanisms have uniformly used male mice to ensure phenotypic consistency and reproducibility of mechanistic findings [35, 36, 37]. Sample size was estimated based on our previous study [38]. Viral vector injection was performed at 8 weeks, followed by cannula implantation and electrode placement at 11 weeks, and KA‐induced neocortical seizure model at 12 weeks. EEG recordings are monitored after KA injection and continued until the EEG recordings were similar to baseline. During the modeling process, mice were anesthetized with isoflurane (3%–4% induction and 1.2% maintenance). The neocortical seizure model was established according to previous studies [39, 40]. After EEG recording, mice were euthanized via cervical dislocation with death confirmed by cardiac arrest and respiratory cessation.

### 2.3 High‐Throughput RNA‐Sequence

The resected human cortical tubers and cultured mouse cortical neurons were used for RNA‐seq (Novogene). Sequencing libraries were generated based on extracted RNA, and differential expression genes (DEGs) were identified using DESeq2 (fold change > 2, FDR < 0.05). Functional enrichment analysis (GO/KEGG) was conducted using ToppGene (adjusted *p* < 0.05). Protein–protein interaction (PPI) networks were built using STRING (confidence score cutoff > 900) and analyzed with NetworkAnalyst, with community detection via InfoMap algorithm and visualization in Cytoscape.

### 2.4 RT‐qPCR

Total RNA was extracted using TRIzol (Invitrogen), quantified by spectrophotometry, and reverse transcribed to cDNA. Detailed sequences of the primers of target DNA are described in Table S1. GAPDH was regarded as the control gene. The amplification conditions were as follows: 95°C for 30 s (1 cycle), followed by 95°C for 5 s (40 cycles) and 60°C for 30 s (1 cycle). Quantitative analysis was performed according to the 2^−ΔΔCt^ method.

### 2.5 Western Blotting and Co‐Immunoprecipitation (Co‐IP)

Proteins were extracted from resected human cortical tubers, mouse cortical tissues, and cultured mouse cortical neurons, with concentration determined by BCA assay. Proteins were separated by 10% SDS‐PAGE, transferred to PVDF membranes, blocked with 5% skim milk, and incubated overnight at 4°C with primary antibodies, including anti‐CD44 (ab243894; 1:500; Abcam), anti‐CASK (ab244393; 1:500; Abcam), anti‐Phospho‐S6 (67898‐1‐Ig; 1:500; proteintech), and anti‐GAPDH (ab181602; 1:1000; Abcam). Membranes were incubated with secondary antibodies for 2 h at room temperature, and bands were visualized by chemiluminescence and quantified using Bio‐Rad Image‐Lab 6.0. For co‐IP, protein extracts from human Epi tubers were incubated overnight at 4°C with anti‐CD44 (ab243894; 1:400; Abcam), anti‐CASK (sc13158; 1:400; Santa Cruz Biotechnology), or immunoglobulin G (IgG; Cell Signaling Technology), followed by protein A/G agarose beads for 2 h at 4°C. Immunoprecipitated complexes were analyzed by western blotting.

### 2.6 IHC and IF

Human and mouse brain tissues were processed into 5‐μm paraffin or 30‐μm cryostat sections. Paraffin sections were deparaffinized, rehydrated, and treated for antigen retrieval. All sections were treated with 3% H_2_O_2_, 0.3% Triton X‐100, and 3% BSA to block endogenous peroxidase and non‐specific binding. For IHC, sections were incubated with anti‐CD44 (ab243894; 1:400; Abcam) and anti‐CASK (ab244393; 1:400; Abcam) overnight at room temperature, followed by secondary antibodies (1:200) and avidin‐biotin‐peroxidase complex, with DAB as the chromogen. For IF, sections were incubated with anti‐CD44 (ab243894; 1:400; Abcam), anti‐NF200 (ab215903; 1:400; Abcam), anti‐GFAP (ab279290; 1:400; Abcam), and anti‐Iba1 (ab283319; 1:400; Abcam), followed by Cy3/488‐conjugated secondary antibodies (1:500, Jackson ImmunoResearch) and DAPI counterstaining. Images were acquired with a Zeiss Axivert microscope, and mean optical density (IHC) and labeling index (IF) were quantified.

### 2.7 ^18^F‐FDG PET Images Processing

^18^F‐FDG PET scans were performed according to Chinese Society of Nuclear Medicine guidelines and our previous study [41]. Patients fasted ≥ 4 h before scanning (blood glucose < 11.1 mmol/L) and were imaged 1 h after injection of 3 MBq/kg FDG in an awake resting state. No seizures occurred during imaging scans. PET images were independently evaluated by two nuclear medicine physicians and two neurosurgeons to identify hypometabolic regions and epileptic foci. Resected tubers were segmented as regions of interest (ROIs) using 3DSlicer, and maximum/mean standardized uptake values (SUV) were extracted for analysis.

### 2.8 Virus Injections

Eight‐week‐old mice were anesthetized with isoflurane (3%–4% induction, 1.2% maintenance). AAV‐hSyn‐CD44‐3xFLAG‐P2A‐EGFP (AAV‐CD44, 1 × 108 TU) was injected into the primary motor cortex (M1) at coordinates: bregma +1.0 mm AP, −1.60 mm ML, −1.60 mm DV (100 nL/min). AAV‐hSyn‐MCS‐3xFLAG‐P2A‐EGFP (AAV‐Con) served as control. The needle was left in place for 5 min post‐injection. Injection accuracy was confirmed by IF, and only mice with correct targeting were included.

### 2.9 Cannula and Electrode Implantation

Three weeks after viral injection, mice were implanted with KA injection guide cannulas and EEG recording electrodes under isoflurane anesthesia (3%–4% induction and 1.2% maintenance). Cortical electrodes were placed bilaterally in M1 (bregma +1.0 mm AP, ±1.60 mm ML, −1.60 mm DV), with reference/ground electrodes over the cerebellum. An injection cannula was attached to the left cortical electrode (0.5 mm above the electrode tip). Mice recovered for 1 week before further experiments.

### 2.10 KA‐Induced Neocortical Seizures Model

Neocortical seizures were induced by intracortical injection of KA (300 ng in 600 nL saline, 500 nL/min) through the pre‐implanted cannula [39, 40]. Seizure severity was scored using the Racine scale: 1, mouth and facial movement; 2, head nodding; 3, forelimb clonus; 4, rearing with forelimb clonus; and 5, rearing and falling with forelimb clonus. Seizure Stage 4–5 was generalized seizures (GS). Seizures Racine scores, seizures onset, average latency to GS and number of GS were recorded and analyzed. Mice failing to develop GS were excluded.

### 2.11 EEG Recording and Analysis

Cortical EEG was recorded using an EEG100C amplifier, with signals filtered (1–500 Hz) and digitized at 1 kHz. Baseline EEG was recorded for several minutes before KA injection. Time‐frequency analysis and power spectral density (δ, θ, α, β, γ bands) were performed using custom code and brainstorm based on Matlab 2021b. Mice were deeply anesthetized with 3%–4% isoflurane and euthanized via cervical dislocation after EEG recording and analysis, with death confirmed by cardiac arrest and respiratory cessation.

### 2.12 Primary Cells Culture

Cortical neurons were isolated from embryonic day 14–16 C57BL/6 mice, digested with 0.05% trypsin–EDTA, and plated at 1 × 105 cells/well on poly‐D‐lysine‐coated 24‐well plates. Cells were cultured in neurobasal medium supplemented with B27 and GlutaMAX for 14 days. For functional experiments, neurons were treated with KA (20 μM) and/or neutralizing anti‐CD44 antibody (10 μg/mL) for 12 h. For CASK knockdown, neurons were transfected with LV‐shCASK or LV‐NC lentivirus. Treated neurons were harvested for RNA‐seq and electrophysiological recordings.

### 2.13 Whole‐Cell Patch‐Clamp Recording

Whole‐cell patch‐clamp recordings were performed using a MultiClamp 700B amplifier on human/mouse brain slices (300 μm) and cultured neurons. Slices were maintained in oxygenated artificial cerebrospinal fluid (ACSF) at 37°C. Spontaneous excitatory postsynaptic current (sEPSC) and spontaneous inhibitory postsynaptic currents (sIPSC) of pyramidal neurons were recorded. Borosilicate glass capillaries were filled with an intracellular solution with a resistance of 3–6 MΩ to record sEPSC and sIPSC. For human brain slices, baseline activity was recorded for 5 min, followed by perfusion with anti‐CD44 antibody (10 μg/mL) for an additional 10 min. For cultured neurons, spontaneous action potentials (APs) were recorded at resting membrane potential during perfusion with hyaluronic acid (HA, 1 μM). All recordings were performed at room temperature using pClamp 9.2 software. Signals were filtered at 5 kHz, digitized at 20 kHz, and analyzed with Clampfit 10.0 software and Mini Analysis Program.

### 2.14 Statistical Analysis

Data are presented as the mean ± SEM for normally distributed data, or median (IQR) for non‐normally distributed data. Statistical analysis was performed with SPSS software. Data were assessed for normality using the Shapiro–Wilk test. For data following a normal distribution, unpaired two‐tailed Student's *t*‐test was used to compare differences between two groups, while one‐way analysis of variance with Tukey's post hoc test was used to compare differences among ≥ 3 groups. For data that do not exhibit a normal distribution, the Mann–Whitney *U*‐test was applied for two‐group comparisons, and the Kruskal–Wallis test with Dunn's post hoc test was applied for comparisons among ≥ 3 groups. Spearman's rank correlation test was performed to evaluate bivariate correlation analyses. ROC curve analysis was used to evaluate the predictive value of CD44 and CASK in identifying Epi tubers. *p* < 0.05 was considered statistically significant.

## 3 Results

### 3.1 Clinical Patient Information

A total of 34 TSC patients were selected, and 25 patients were finally enrolled after excluding 4 without intractable epilepsy, 4 with insufficient clinical data, and 1 with imaging contraindications (Figure S1, Table S2). We collected 57 cortical tubers (37 Epi, 20 non‐Epi), identified by imaging features and SEEG recordings (Figure 1A–C). All patients achieved seizure freedom after Epi tuber resection. Non‐Epi tuber resection was limited due to clinical and ethical considerations, resulting in fewer samples. Group differences are summarized in Table S3.

**FIGURE 1:**
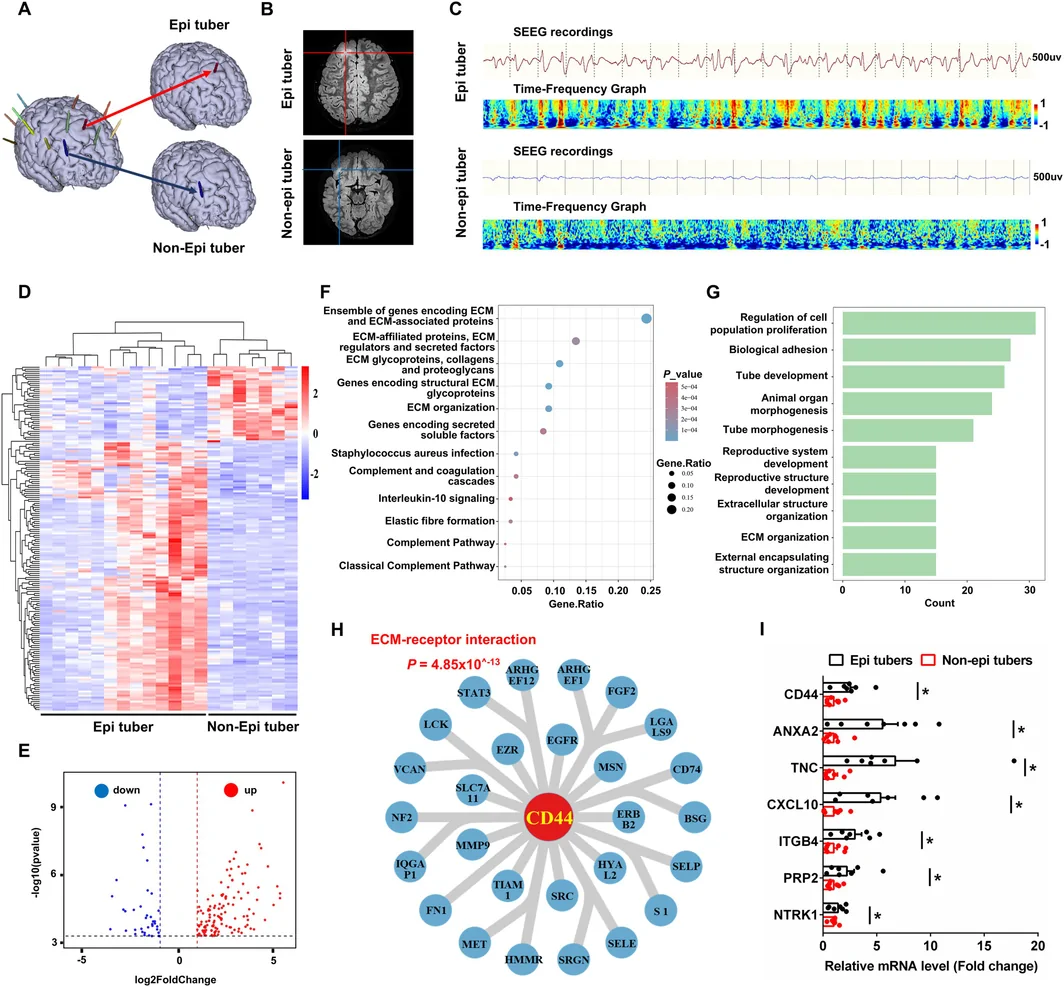
CD44 participated in the epileptogenesis of Epi tubers in patients with TSC included. (A–C) Preoperative location of Epi and non‐Epi tubers. (D) Hierarchical clustering heatmap for DEGs between Epi tubers (*n* = 13) and non‐Epi tubers (*n* = 7). (E) Volcano Plot of upregulated genes and downregulated genes between Epi and non‐Epi tubers. (F) Enriched GO pathways in biological process. (G) Enriched KEGG pathways in biological process. (H) PPI network analyses screened out CD44 as the Hub gene. (I) qRT‐PCR was performed to verify the results of RNA‐seq. *n* = 7 for each group. Statistical tests used for analysis were two‐tailed unpaired *t*‐tests. Data were shown as means ± SEM. *p < 0.05. Epi, epileptogenic; GO, Gene ontology; KEGG, Kyoto Encyclopedia of Genes and Genomes; PPI, protein–protein interaction.

### 3.2 CD44 Participated in the Epileptogenesis of Cortical Tubers in Patients With TSC

RNA‐seq of 13 Epi and 7 non‐Epi tubers identified 228 DEGs (150 upregulated, 78 downregulated) (Figure 1D,E). GO and KEGG enrichment revealed predominant involvement of extracellular matrix (ECM) organization and cell adhesion pathways (Figure 1F,G). PPI network analysis identified CD44 as the hub gene in the ECM‐receptor interaction community (Figure 1H). RNA‐seq results were validated by RT‐qPCR for 7 randomly selected DEGs (ANXA2, TNC, CXCL10, ITGB4, CD44, PRP2, and NTRK1) (Figure 1I).

### 3.3 Increased CD44 Expression in Epi Tubers Compared With Non‐Epi Tubers

CD44 expression was significantly higher in Epi tubers (*n* = 37) than non‐Epi tubers (*n* = 20) by western blotting and IHC (Figure 2A–D). To assess specificity, we analyzed CD44 expression in TLE (*n* = 5), FLE (*n* = 4), and control (*n* = 4) specimens (Tables S4 and S5). No significant difference in CD44 expression was detected in these groups (Figure S2), indicating relative specificity for TSC. Immunofluorescence results demonstrated that CD44 upregulation occurred primarily in neurons, not astrocytes or microglia (Figure 2E,F).

**FIGURE 2:**
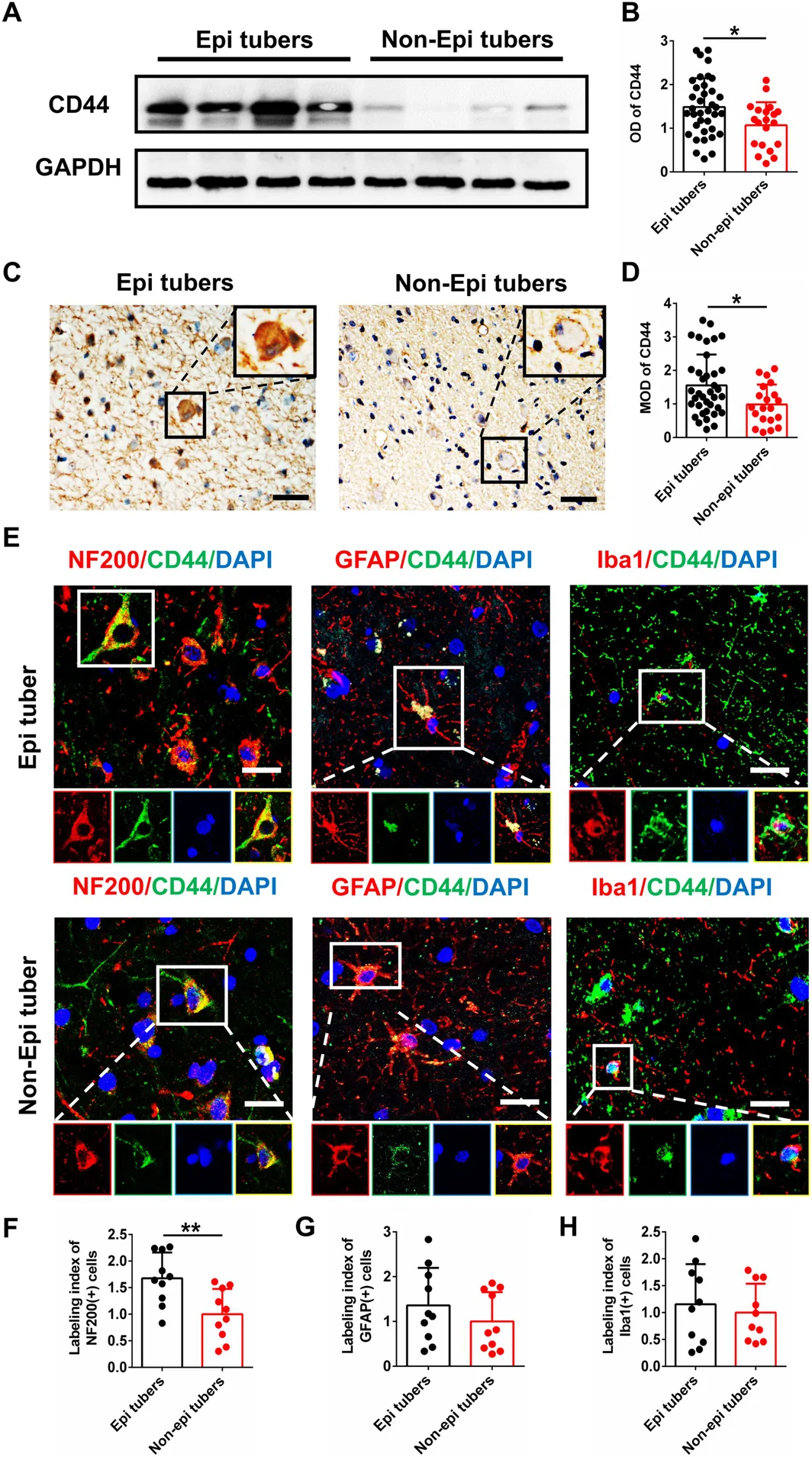
Increased CD44 expression in resected human Epi tubers compared with non‐Epi tubers. (A) Western blot analysis of CD44 in resected Epi and non‐Epi tubers from patients with tuberous sclerosis complex. (B) Western blot analysis showed that the optical density of CD44 was increased in Epi tubers (*n* = 37) compared with non‐Epi tubers (*n* = 20). (C) Immunostaining of CD44 in resected Epi and non‐Epi tubers. Scale bar, 50 μm. (D) Immunostaining analysis showed that the MOD of CD44 was increased in Epi tubers (*n* = 37) compared with non‐Epi tubers (*n* = 20). (E) Immunofluorescence analysis showed the distribution of CD44 in neurons, astrocytes and microglia. (F–H) The Labeling index of co‐labeled cell in immunofluorescence showed CD44 expression mainly upregulated in neurons (F), but not in astrocytes (G) and microglia (H). *n* = 10 for each group. Scale bar, 25 μm. Statistical analysis was conducted with two‐tailed unpaired Student's *t*‐test. Data are shown as means ± SEM. *p < 0.05, **p < 0.01. Epi, epileptogenic.

### 3.4 Correlation Between CD44 Expression and Clinical Characteristics of Epi Tubers

CD44 expression positively correlated with seizure frequency (*r* = 0.4058, *p* < 0.05) and duration (*r* = 0.3941, *p* < 0.05), but not with tuber size (Figure S3A–C). Consistent with previous findings [41], Epi tubers had significantly lower SUVmean and SUVmax than non‐Epi tubers (Figure S4A,B). CD44 expression was negatively correlated with SUV values in Epi tubers (SUVmean: *r* = −0.4345, *p* < 0.01; SUVmax: *r* = −0.3705, *p* < 0.05), non‐Epi tubers (SUVmean: *r* = −0.5426, *p* < 0.05; SUVmax: *r* = −0.5538, *p* < 0.05), and all tubers combined (Figures S3E–H and S4C,D), suggesting that CD44 upregulation had a potential association with the epileptogenesis of cortical tubers.

### 3.5 CD44 Regulates the Susceptibility to Neocortical Seizures in Mouse Model

TSC is a common cause of neocortical seizures, so we established the neocortical seizures model according to previous study to investigate the effect of CD44 on the neocortical seizures of TSC [39]. The results showed increased CD44 expression consistent with human Epi tubers (Figure S5A,B), validating that this model is suitable for the present study. We injected AAV‐CD44 or control AAV into the left primary motor cortex (M1) of 8‐week‐old mice, followed by cannula/electrode implantation at 11 weeks and KA induction at 12 weeks (Figure 3A). Immunostaining confirmed efficient and specific CD44 overexpression in M1 (Figure 3B–E). CD44 overexpression significantly increased δ, θ, and γ oscillations during seizures (Figure 3F,G). Behaviorally, AAV‐CD44 mice exhibited higher maximum Racine scores, more GS, shorter seizure onset, and shorter latency to first GS compared with controls (Figure 3H–L).

**FIGURE 3:**
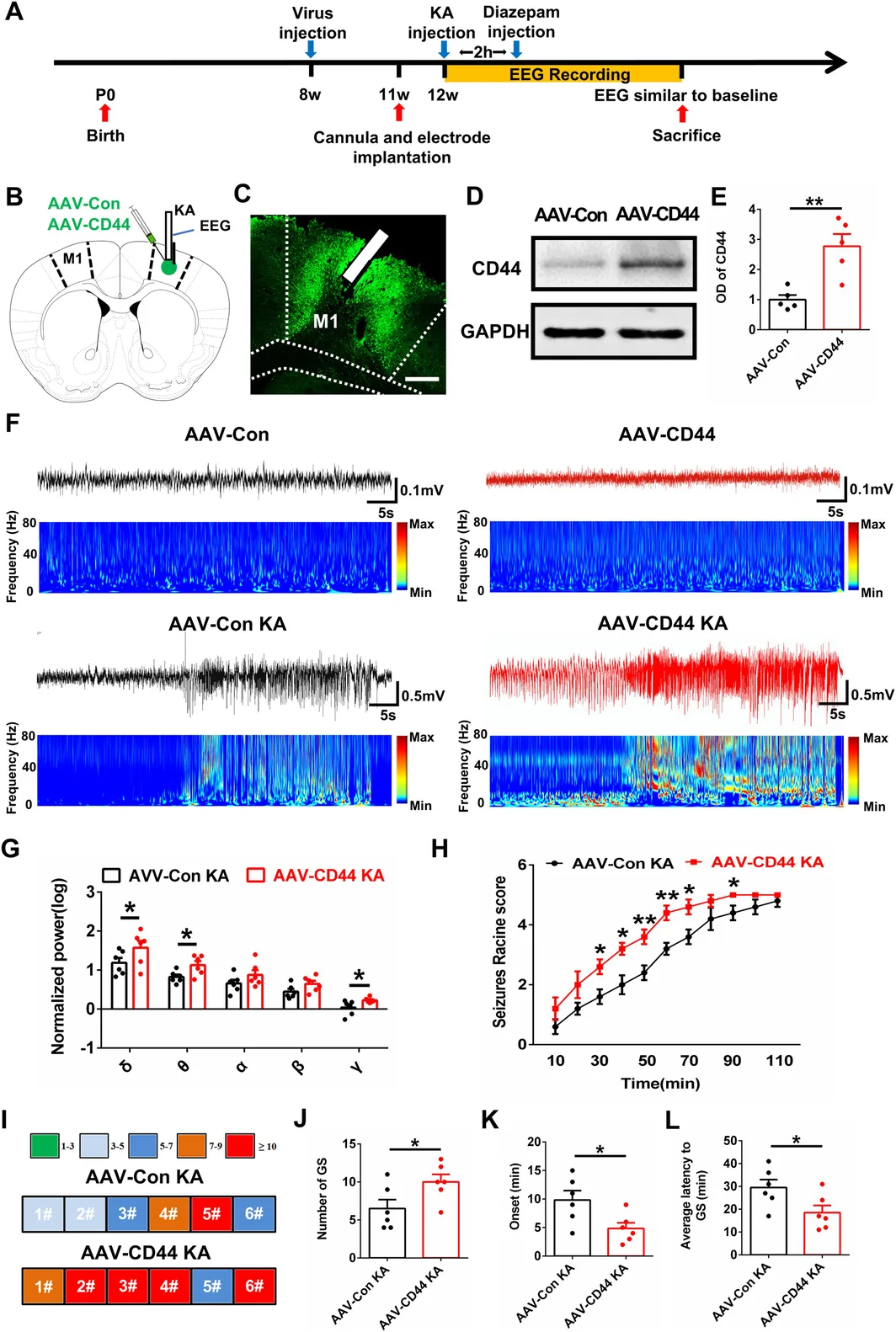
CD44 activation increased the susceptibility to neocortical seizures. (A) Schematic diagram of the experimental design. (B and C) Representative image of GFP‐positive cells in the primary motor cortex after AAV‐CD44 and AAV‐Con injection. Scale bar, 50 μm. (D) Western blot of CD44 in the primary motor cortex of mice injected with AAV‐CD44 and AAV‐Con. (E) Western blot analysis showed that OD of CD44 was increased in the primary motor cortex injected with AAV‐CD44 than AAV‐Con. *n* = 5 for each group. Statistical tests used for analysis were two‐tailed unpaired *t*‐tests. (F) Representative basal and epileptic EEG recordings and time‐frequency diagram from the primary motor cortex of mice. (G) Spectral analysis of epileptic EEG recordings (*n* = 6 for each group). AAV‐CD44 injection increased *δ*, *θ*, and *γ* oscillations in the epileptic primary motor cortex of mice. (H) Seizure behavioral score in mice (*n* = 5 for each group). (I–L) Number of GS (I and J), seizures onset (K), and average latency to GS (L) in mice (*n* = 6 for each group). AAV‐CD44 injection increased seizure behavioral scores and number of GS, and decreased seizures onset and average latency to GS in epileptic mice. Statistical tests used for analysis were two‐tailed unpaired *t*‐tests. Data were shown as means ± SEM. *p < 0.05, **p < 0.01. GS, generalized seizures; OD, optical density.

### 3.6 CD44 Regulated Cortical Pyramidal Neuronal Excitability in Both Mouse and Human Brain Slices

Given that CD44 was primarily upregulated in neurons (Figure 2F), we investigated its effect on E/I balance using whole‐cell patch‐clamp. In mouse brain slices, CD44 overexpression significantly increased sEPSC frequency but not amplitude in cortical pyramidal neurons, with no effect on sIPSC (Figure 4A–J). In human Epi tuber slices, pyramidal neurons showed increased sEPSC frequency and decreased sIPSC frequency compared with non‐Epi tubers. Perfusion with anti‐CD44 neutralizing antibody significantly reduced the elevated sEPSC frequency but did not rescue the decreased sIPSC frequency (Figure 4K–T). Notably, both epileptic mouse and human brain slices showed increased sEPSC frequency and decreased sIPSC frequency compared with non‐epileptic slices, but the amplitude of sEPSC and sIPSC was not different, indicating the reliability of this neocortical seizure model in studying the E/I balance of cortical pyramidal neurons in Epi and non‐Epi tubers (Figure S6A,B).

**FIGURE 4:**
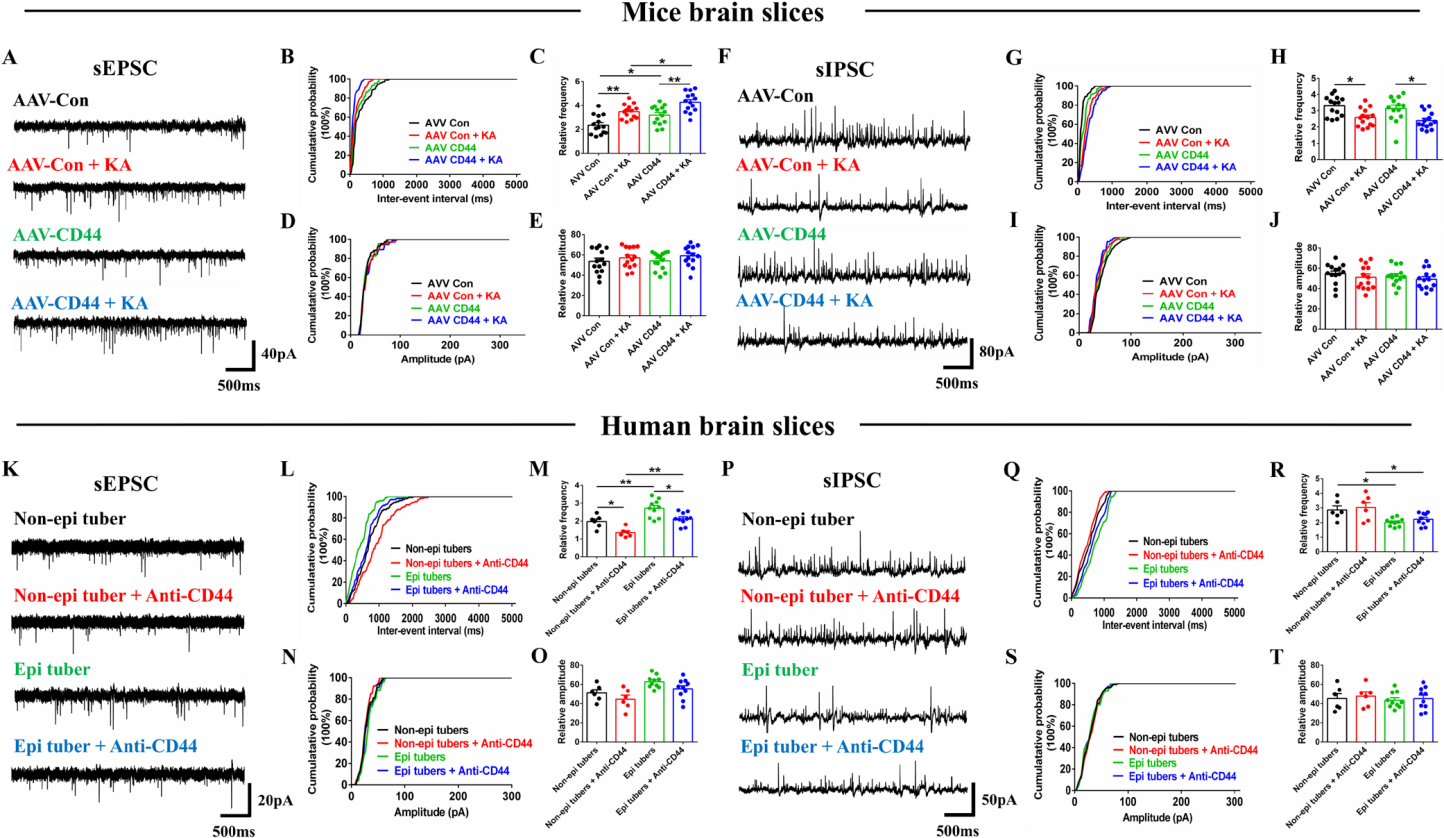
CD44 regulated the pyramidal neuronal E/I imbalance in human and mice epileptic cortex. (A) Representative sEPSCs trace of cortical pyramidal neurons from mice brain slices. (B–E) Cumulative probability distribution and statistical comparison of sEPSC frequency and amplitude. The sEPSC frequency of cortical pyramidal neurons was significantly increased in epileptic mice compared with controls. AAV‐CD44 injection especially aggravated the increased sEPSC frequency in epileptic cortical pyramidal neurons. But there were no differences in the sEPSC amplitude of the four groups. (F) Representative sIPSCs traces of cortical pyramidal neurons from mice brain slices. (G–J) Cumulative probability distribution and statistical comparison of sIPSC frequency and amplitude. There were no differences in sIPSC frequency and amplitude of the four groups. *n* = 14 for each group. (K) Representative sEPSCs trace of cortical pyramidal neurons from human Epi and non‐Epi tubers. (L–O) Cumulative probability distribution and statistical comparison of sEPSC frequency and amplitude. The sEPSC frequency of cortical pyramidal neurons was significantly increased in Epi tubers compared with non‐Epi tubers, and CD44 monoclonal antibodies rescued the increased sEPSC frequency. But there were no differences in the sEPSC amplitude of the three groups. (P) Representative sIPSCs trace of cortical pyramidal neurons from human Epi and non‐Epi tubers. (Q–T) Cumulative probability distribution and statistical comparison of sIPSC frequency and amplitude. There was decreased sIPSC frequency of cortical pyramidal neurons in Epi tubers compared with non‐Epi tubers, but CD44 monoclonal antibodies perfusion did not affect the decreased sIPSC frequency. There were no differences in sIPSC amplitude of the three groups. *n* = 6 cells of non‐Epi tubers from 3 patients, *n* = 10 cells of Epi tubers from 5 patients, and *n* = 10 cells of Epi tubers perfused with CD44 monoclonal antibodies from 5 patients. Significance was calculated using ANOVA, followed by Tukey's test. Data were shown as means ± SEM. *p < 0.05, **p < 0.01. Epi, epileptogenic; EPSC, excitatory postsynaptic current; IPSC, inhibitory postsynaptic current.

### 3.7 CASK Participates in CD44‐Regulated Cortical Neuronal Excitability

To identify downstream targets of CD44, we established an in vitro hyperexcitable neuronal model by treating primary cortical neurons with KA, which upregulated CD44 expression; this effect was reversed by anti‐CD44 neutralizing antibody (Figure S7A,B).

RNA‐seq identified 11,133 DEGs (5630 upregulated and 5503 downregulated genes) regulated by CD44 (Figure 5A,B). GO enrichment analysis was performed on those DEGs and showed that they were mainly involved in regulating cell division, positive regulation of catabolic processes, proteasomal protein catabolic processes, cell cycle phase transition, and neuron‐to‐neuron synapses (Figure 5C). KEGG analysis was further performed and showed that the DEGs were mainly enriched in the interaction of neurexins and neuroligins at synapses, neuronal system, and PPIs at synapses (Figure 5D). These results could explain the changes in sEPSC frequency in cortical neurons. PPI network analysis identified CASK as the hub gene in the glutamatergic synapse community (Figure 5E). RNA‐seq results were confirmed by verifying hub gene (HOMER1, GRIN1, CASK, CAMK2A, LIN7C, and NRXN3) expression via RT‐qPCR (Figure 5F) and CASK expression via western blotting (Figure 5G,H).

**FIGURE 5:**
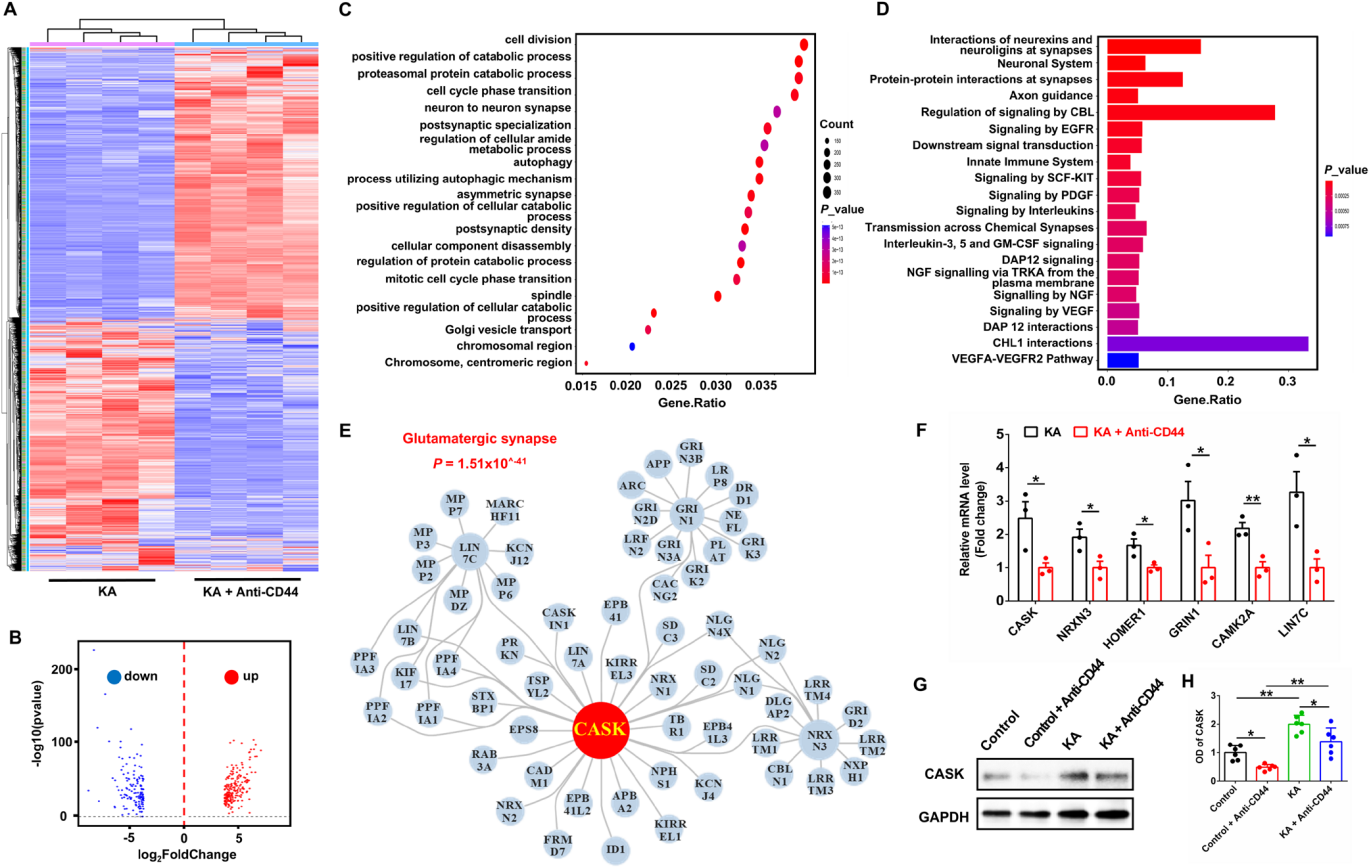
CASK participated in CD44‐regulated cortical neuronal excitability. (A) Hierarchical clustering heatmap showed DEGs regulated by CD44 in cultured cortical hyper‐excitable neurons. (B) Volcano Plot showed upregulated and downregulated DEGs. (C) Enriched GO pathways in biological process. (D) Enriched KEGG pathways in biological process. (E) PPI network analyses screened out CASK as the hub gene. (F) qRT‐PCR was performed to verify the results of RNA‐seq. *n* = 3 for each group. Statistical tests used for analysis were two‐tailed unpaired *t*‐tests. (G) Western blot of CASK in cultured primary cortical neurons treated with KA and CD44 monoclonal antibodies. (H) Western blot analysis showed KA induced the increased OD of CASK and CD44 monoclonal antibodies rescued the increased CASK caused by KA in primary cortical neurons. *n* = 6 for each group. Significance was calculated using ANOVA, followed by Tukey's test. Data were shown as means ± SEM. *p < 0.05, **p < 0.01. GO, Gene ontology; KEGG, Kyoto Encyclopedia of Genes and Genomes; PPI, protein–protein interaction.

### 3.8 CD44–CASK Pathway Regulates the Cortical Neuronal Excitability and Epileptogenesis of Epi Tubers

To confirm that CD44 regulates neuronal excitability via CASK, we recorded APs in cultured cortical neurons transfected with LV‐shCASK or LV‐NC (Figure 6A). LV‐shCASK effectively downregulated CASK expression (Figure S8A–C). CASK knockdown significantly reduced neuronal firing frequency and increased AP threshold, effects that were reversed by the CD44 agonist hyaluronic acid (HA) (Figure 6B,C). CASK expression was significantly higher in Epi tubers than non‐Epi tubers (Figure 6D–G). Similar to CD44, CASK expression was positively correlated with seizure frequency (*r* = 0.7081, *p* < 0.05) and duration (*r* = 0.7126, *p* < 0.05), and negatively correlated with SUVmean (*r* = −0.8597, *p* < 0.01) and SUVmax (*r* = −0.8336, *p* < 0.01) (Figure 6H–K). CD44 and CASK expression were strongly positively correlated (*r* = 0.8532, *p* < 0.01) (Figure 6L), and co‐IP confirmed their direct interaction in human Epi tubers (Figure 6M). Additionally, phospho‐S6 (p‐S6, a marker for mTORC1 activity) and its downstream transcription factors STAT3, HIF‐1α, and NF‐κB were all upregulated in Epi tubers (Figure S10A,B,G–I). p‐S6 was also upregulated in KA‐treated mouse cortex (Figure S10C,D), and p‐S6 levels were positively correlated with both CD44 and CASK expression across Epi tuber samples (Figure S10E,F).

**FIGURE 6:**
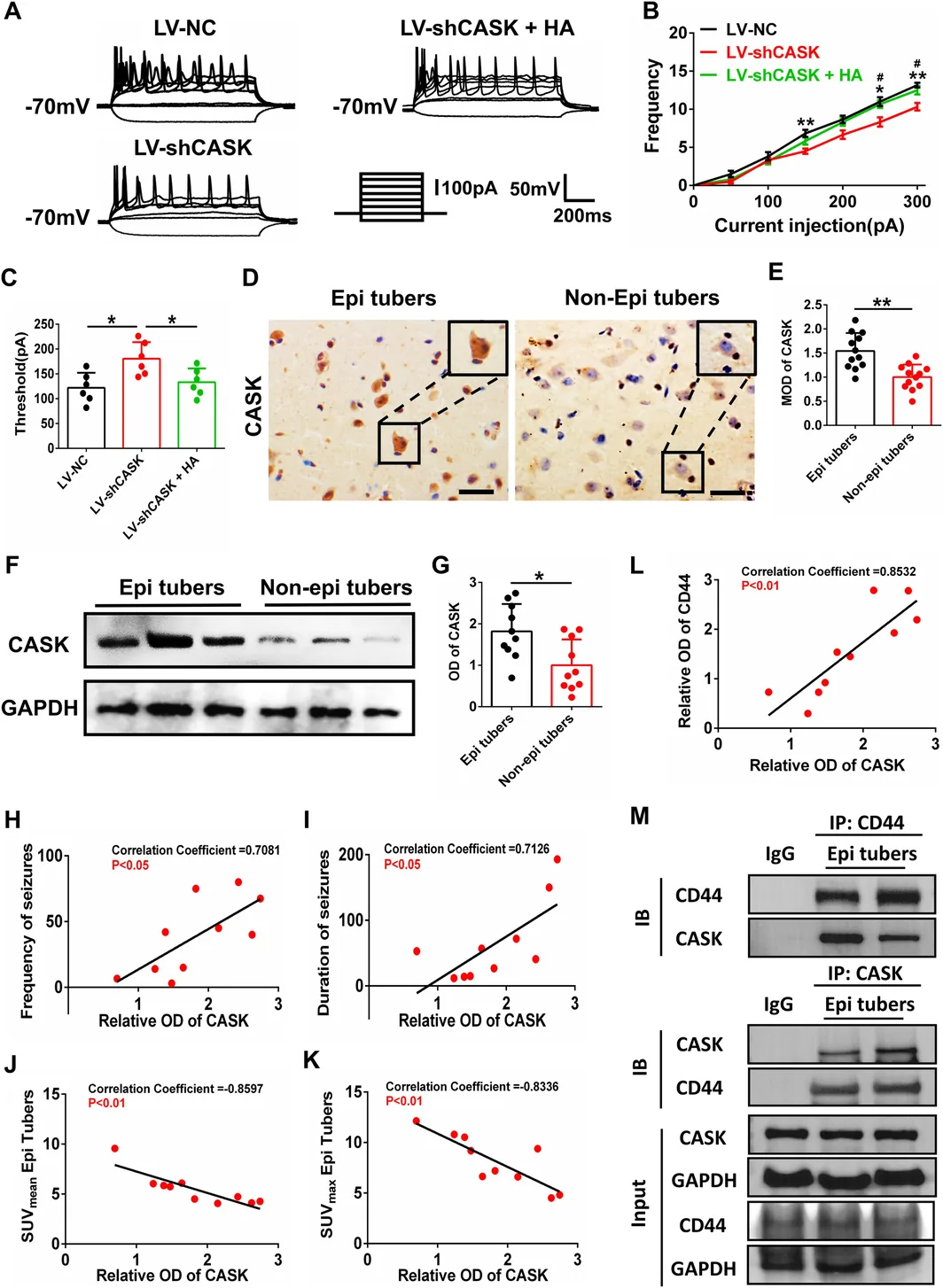
CD44 regulated the cortical neuronal excitability and epileptogenesis of human Epi tubers through the CASK pathway. (A) Representative trace showing action potential spiking in cortical neurons. (B) APs frequency induced by increasing depolarizing currents in cultured cortical neurons. (C) Spike threshold for cultured cortical neurons. LV‐shCASK neurons showed a lower firing frequency in response to injection currents and greater AP threshold than LV‐NC neurons, which were reversed by HA. *n* = 6 for each group. Significance was calculated using ANOVA, followed by Tukey's test. * represents LV‐NC versus LV‐shCASK; *p < 0.05, **p < 0.01; # represents LV‐shCASK versus LV‐shCASK + HA; #*p* < 0.05. (D) Immunostaining of CASK in resected Epi and non‐Epi tubers. Scale bar, 50 μm. (E) Immunostaining analysis showed that MOD of CASK was increased in Epi tubers compared to non‐Epi tubers. *n* = 12 for each group. Statistical tests used for analysis were two‐tailed unpaired *t*‐tests. (F) Western blot of CASK in resected Epi tubers and non‐Epi tubers from patients with TSC. (G) Western blot analysis showed that OD of CASK was increased in Epi tubers than non‐Epi tubers. *n* = 10 for each group. Statistical tests used for analysis were two‐tailed unpaired *t*‐tests. Data were shown as means ± SEM. *p < 0.05, **p < 0.01. (H and I) Spearman rank correlation of CASK expression (OD of CASK) with seizure frequency (H) and seizure duration (I). CASK expression was positively correlated with seizure frequency (correlation coefficient = 0.7081, *p* < 0.05) and seizure duration (correlation coefficient = 0.7126, *p* < 0.05). (J and K) Spearman rank correlation of CASK expression with SUVmean (J) and SUVmax (K) of Epi tubers. CASK expression was negatively correlated with SUVmean (correlation coefficient = −0.8597, *p* < 0.01) and SUVmax (correlation coefficient = −0.8336, *p* < 0.01) in Epi tubers. *n* = 10 for each group. (L) Spearman rank correlation of CASK expression with CD44 expression; there was a positive correlation between CASK expression and CD44 expression (correlation coefficient = 0.8532, *p* < 0.01). (M) Co‐IP analysis demonstrated the interactions between CD44 and CASK in Epi tubers. Characterization of interactions between CD44 and CASK, and reverse validation of the interaction of CASK with CD44 in Epi tubers by Co‐IP. Statistical tests used for analysis were Spearman's rank correlation tests. IgG immunoprecipitate was used as a negative control. AP, action potential; Epi, epileptogenic; IP, immunoprecipitation.

### 3.9 CD44–CASK Pathway Acts as a Potential Biomarker in Identifying Epi Tubers and Predicting Surgical Outcomes in TSC

ROC curve analysis showed that CD44 (AUC = 0.82, *p* = 0.015), CASK (AUC = 0.78, *p* = 0.034), and their combination (AUC = 0.90, *p* = 0.003) effectively distinguished Epi from non‐Epi tubers (Figure S9A–C), with the combined marker showing the best performance. We further evaluated the prognostic value of these markers in patients with a single Epi tuber (Table S6). All patients were seizure‐free at 1‐year follow‐up. At 5‐year follow‐up, the combined CD44–CASK marker had an AUC of 0.78 for predicting seizure recurrence, which improved to 0.86 at 8‐year follow‐up. These results indicate that activation of the CD44–CASK pathway could be a potential biomarker for identifying Epi tubers from among all cortical tubers and predicting surgical outcomes in patients with TSC.

## 4 Discussion

Previous studies have reported that the ECM adhesion protein CD44 is upregulated during brain injury and may be a key epilepsy‐associated protein [22, 26]. Our study demonstrated that increased CD44 contributed to cortical neuronal hyperexcitability and tuber epileptogenesis in TSC. CASK, a known gene associated with focal cortical malformations including TSC [42], was found to be upregulated in Epi tubers and activated by CD44 in hyperexcitable neurons and cortical seizures of TSC. CD44 and CASK expression levels were closely correlated with clinical epileptic characteristics of Epi tubers, enabling identification of Epi tubers and prediction of surgical outcomes. These results indicate that CD44 is a potential therapeutic target for TSC‐related DRE.

ECM is an important regulator of neuronal excitability and synaptic plasticity [43, 44]. ECM alterations can disrupt E/I balance, trigger excessive neuronal synchronization, and contribute to epileptogenesis [44, 45]. Pathological ECM remodeling is a potential inducing factor of epileptogenesis, inducing hippocampal sclerosis and long‐term functional changes in TLE [23, 46, 47]. HA, the main ECM component, supports neuronal activity, regulates synapse formation, and maintains epileptiform hypersynchrony [20, 22]. TSC is a typical cause of malformation of cortical development accompanied by DRE, indicating the specific value of the ECM and HA in the epileptogenesis of TSC. Our RNA‐seq analysis of human Epi and non‐Epi tubers confirmed that DEGs were primarily enriched in ECM‐related pathways, highlighting the importance of investigating ECM and HA signaling in the epileptogenesis of TSC.

HA signaling via its receptor CD44 regulates multiple cellular processes in neurological disorders [25, 48]. Elevated CD44 expression has been observed in various central nervous system (CNS) insults, including ischemia, trauma, and neurodegenerative diseases [48, 49, 50, 51], as well as in KA‐ and pilocarpine‐induced epilepsy models [24, 27]. Our RNA‐seq results identified CD44 as the hub gene in the ECM‐receptor interaction community, with significantly higher expression in human Epi tubers and mouse epileptic cortex. In addition, CD44 expression also positively correlated with seizure frequency and duration in TSC patients, suggesting its involvement in TSC epileptogenesis. CD44 is essential for CNS development, regulating stem cell proliferation, neuronal maturation, and neocortical development [27]. Interestingly, no significant difference in CD44 expression was detected in temporal lobe epilepsy and frontal lobe epilepsy compared with controls, indicating that CD44 upregulation is relatively specific to TSC‐associated epilepsy. Our observation of increased CD44 in dysplastic neurons of human Epi tubers suggests that CD44 overexpression may contribute to both cortical dysplasia formation and epileptogenesis in TSC.

TSC is an autosomal dominant genetic syndrome caused by TSC1 or TSC2 gene mutations, resulting in overactivation of the mTOR signaling pathway. Previous studies have provided evidence for the regulatory link between mTORC1 and CD44 [52]. mTORC1 hyperactivation drives CD44 upregulation through multiple downstream transcription factors, including STAT3, HIF‐1α, and NF‐κB, all of which are hyperactivated in Epi tubers and have confirmed binding sites in the CD44 regulatory region [53, 54, 55]. And we confirmed upregulated phospho‐S6 expression in Epi tubers are positively correlated with CD44 expression in Epi tubers. Notably, while CD44 has been implicated in neuroinflammation [26], studies in epilepsy models have shown that CD44 upregulation does not coincide with microglial activation, suggesting a primary role in neuronal E/I balance rather than immune responses [27, 43]. Our finding that CD44 was upregulated in neurons but not astrocytes or microglia further supports this conclusion. E/I imbalance is the key trigger underlying neuronal hyperexcitability and epileptic seizures. Previous studies have reported that CD44 influences the excitability of sensory neurons following injury, and that increased CD44 expression is associated with TLE [24, 27, 56]. We found that CD44 activation in mouse motor cortex increased seizure susceptibility and sEPSC frequency in cortical neurons, while anti‐CD44 neutralizing antibody rescued the E/I imbalance in human Epi tuber slices. It is notable that pyramidal neurons are the typical excitatory neurons, and dysmorphic pyramidal neurons displayed low‐to‐mild Epi potential [57]. Roszkowska et al. [58] demonstrated that CD44 localizes to mature synapses and regulates excitatory synaptic transmission by modulating actin cytoskeleton dynamics, explaining its specific effect on sEPSC frequency.

The CASK gene encodes a calcium/calmodulin‐dependent serine protein kinase involved in synaptic interaction, protein trafficking, and gene expression regulation and is associated with a variety of conditions, such as intellectual disability, cerebellar hypoplasia, and epileptic encephalopathy [42, 59]. We found significantly higher CASK expression in human Epi tubers, which positively correlated with seizure severity, supporting the role of CASK in epileptic encephalopathy. CASK regulates neurotransmitter release by interacting with voltage‐gated calcium channels and NMDA receptors [59, 60], and CASK knockdown in primary neurons reduced action potential firing frequency and increased AP threshold, effects reversed by the CD44 agonist HA. Clinical studies have reported CASK mutations causing early‐onset seizures and cortical malformations [42, 61], consistent with our finding that increased CASK expression in dysplastic neurons participates in TSC epileptogenesis. In addition, RNA‐seq analysis identified CASK as a downstream target of CD44 that regulates cortical neuronal excitability, and increased CASK and CD44 expression were positively correlated. We confirmed this interaction between CD44 and CASK in Epi tubers using co‐immunoprecipitation. These results further support the Epi effect of the CD44–CASK signaling pathway in Epi tubers.

Previous studies on CD44 in epilepsy have yielded conflicting results, with some reporting upregulation [29, 62] and others downregulation [27] in epileptic animal models. Peripheral blood CD44 upregulation has also been observed in epilepsy patients [63], but this does not directly reflect brain tissue expression. Our study using resected human Epi and non‐Epi tubers provides more reliable evidence for CD44 upregulation in TSC epileptogenesis.

Precise localization of Epi tubers among multiple lesions remains the major challenge in TSC epilepsy surgery, as seizures often arise from multiple independent foci [7, 64]. Precise localization of Epi tubers among multiple cortical tubers determines the outcomes of surgical resection and prognosis [14]. Clinical markers for identifying Epi tubers have been widely reported, including clinical characteristics, EEG recordings, and imaging examinations [10, 19, 65]. Notably, ^18^F‐FDG PET is commonly used in the presurgical assessment of patients with TSC by identifying hypometabolic seizure onset tubers, particularly when MRI and scalp EEG cannot localize the Epi zone or are discordant [66]. However, the molecular biomarkers used to identify Epi tubers from multiple cortical tubers in TSC are still scarce. Dysmorphic neurons in TSC are regarded as ‘epileptic generators’, and a previous study suggested that a high concentration of dysmorphic neurons in the center of Epi tubers forms the basis of their imaging appearance [67]. Therefore, the present study explored whether increased CD44 expression in dysmorphic neurons could be used as a molecular biomarker to identify the “epileptic generators” in TSC. A negative correlation was found between CD44 and CASK expression and SUV, indicating that hypometabolism in the center of Epi tubers as observed on ^18^F‐FDG PET may be caused by increased CD44 expression. Furthermore, the potential value of CD44 and CASK expression in locating Epi tubers in TSC was evaluated, and the AUC of the ROC curve demonstrated that both CD44 and CASK expression had predictive value. These results provide additional, complementary molecular biomarkers for identifying Epi tubers from multiple cortical tubers in TSC.

Notably, anti‐CD44 monoclonal antibodies have already demonstrated safety and efficacy in Phase 1/2 clinical trials for various cancers (NCT01358903, NCT01641250, NCT02046928). Our finding that anti‐CD44 antibody rescued sEPSC abnormalities in human Epi tuber slices supports the potential repurposing of these agents for TSC‐related DRE, though further preclinical and clinical studies are required.

This present study has several limitations. First, the sample size of resected cortical tubers is relatively small. TSC is a rare neurological disorder, in which 63%–78% of patients develop focal seizures or epileptic spasms. Our 57 tubers (37 Epi, 20 non‐Epi) meet the minimum statistical power requirement (> 0.8) and the acceptable Epi:non‐Epi ratio (37:20) [68]. It is worth noting that resection of non‐Epi tubers is more stringent than that of Epi tubers due to clinical and ethical considerations, resulting in fewer non‐Epi tubers than Epi tubers used in the present study. Future larger cohorts are needed to validate the specificity and mechanism of CD44–CASK up‐regulation in TSC‐associated epilepsy. Second, current animal models have not replicated the specific pathological structure of cortical tubers in TSC. Previous studies reported that TSC1/2 deletion, RhebCA electroporation and KA injection could induce a mouse model of TSC, and these TSC models displayed partial disease features of TSC, including cell misplacement, increased soma size and neuronal dysmorphogenesis [39, 69, 70, 71, 72]. However, the specific pathology of cortical tubers was not replicated in these animal models. Consequently, the present study investigated the role of CD44 in neuronal E/I balance using resected cortical tubers from patients with TSC. Furthermore, the KA‐induced neocortical epilepsy model was used to assess CD44's role in cortical seizure susceptibility in TSC and partly validate its involvement in the epileptogenesis of Epi tubers based on previous studies [39, 73]. Third, CD44 detection currently relies on post‐resection tissue. Future development of CD44‐specific PET tracers will enable noninvasive in vivo localization of Epi tubers.

## 5 Conclusion

This study showed that increased expression of CD44 in human Epi tubers was correlated with poor clinical features and decreased SUVs on ^18^F‐FDG PET, and upregulated CD44 expression aggravated mouse neocortical seizures. In vivo and in vitro electrophysiology results suggested that CD44 regulated neocortical seizures in mice and cortical neuronal E/I imbalance in human and mouse epileptic brain slices. Furthermore, the CASK pathway was activated by increased CD44 to regulate cortical neuronal excitability and epileptic clinical features in human Epi tubers. The present findings suggest that activated CD44–CASK acts as a trigger for epileptogenesis and could serve as a potential biomarker for identifying Epi tubers and predicting surgical outcomes, which could provide a novel treatment target for TSC‐induced DRE and a locational biomarker for Epi tubers in patients with TSC.

## Author Contributions

Shiyong Liu, Xiaolin Yang, Hui Yang, and Zhongke Wang conceived, executed and supervised the study. Zhongke Wang, Kaixuan Huang, Xingye Liu, and Yang Li performed statistical analyses and prepared the first draft of the manuscript. Shujing Li, Jie Li, Hu Li, and Yali Cheng collected the patient's clinical data. Kaixuan Huang, Jingze Wang, Zeng He, Limeng Sun, and Ruotong Ruan performed molecular biological experiments and bioinformatics analysis. Zhongke Wang, Jie Li and Yang Li performed the in vivo electrophysiology. Zhongke Wang and Xiaolin Yang performed the in vitro electrophysiology. Zhonghong Liu, Shengqing Lv, Chunqing Zhang, Hui Yang, and Shiyong Liu performed comprehensive preoperative assessment and epilepsy surgery. Correspondence and requests for materials should be addressed to Shiyong Liu, Xiaolin Yang and Zhongke Wang. All authors read and approved the final version of the manuscript.

## Funding

This work was supported by the National Natural Science Foundation of China (82571660), Chongqing Medical Young Talent Project (YXQN202403), Chongqing medical scientific research project (2026QNXM034), and Special Project for Clinical Research of the Second Affiliated Hospital of Army Medical University (2024F033).

## Ethics Statement

The study was approved by the Medical Ethics Committee of Xinqiao Hospital (Chongqing, China; approval no. 395‐01/2022), and we also analyzed resected specimens from patients with temporal lobe epilepsy (TLE), frontal lobe epilepsy (FLE) and controls (ethical approval no. 362‐01/2024), and the study of animals was approved by Ethics Committee of Army Medical University (approval no. AMUWEC20224423). All procedures were performed according to the guidelines of the Declaration of Helsinki of the World Medical Association and our institutional review board.

## Conflicts of Interest

The authors declare no conflicts of interest.

## Supporting information


**Figure S1:** Flowchart of patient inclusion and exclusion criteria. A total of 34 patients with tuberous sclerosis complex presented to Xinqiao hospital from June 2017 to January 2023, and four patients without intractable epilepsy, four patients without sufficient clinical information and one patient with imaging contraindications were excluded. In total, 25 patients were enrolled in the current study. Epi, epileptogenic.
**Figure S2:** CD44 expression in patients with TLE and FLE, and controls. (A) Western blot analysis of CD44 in TLE, FLE and controls. (B) Western blotting showed no significance difference in the optical density values of CD44 in TLE (*n* = 5), FLE (*n* = 4) or controls (*n* = 4). FLE, frontal lobe epilepsy; TLE, temporal lobe epilepsy.
**Figure S3:** Correlation analyses of CD44 expression and clinical features of Epi tubers. (A‐C) Spearman rank correlation analysis between CD44 expression (optical density of CD44) and size (A), seizures frequency (B) and seizures duration (C) of Epi tubers. There was no correlation between CD44 expression and size of Epi tubers (correlation coefficient = 0.1783, p = 0.29), but CD44 expression was positively correlated with seizure frequency (correlation coefficient = 0.4058, *p* < 0.05) and seizures duration (correlation coefficient = 0.3941, *p* < 0.05). (D) Examples of preoperative localization of Epi and non‐Epi tubers on imaging. (E and F) Spearman rank correlation analysis of CD44 expression with SUVmean (E) and SUVmax (F) of Epi tubers. CD44 expression was negatively correlated with SUVmean (correlation coefficient = −0.4345, *p* < 0.01) and SUVmax (correlation coefficient = −0.3705, *p* < 0.05) in Epi tubers. (G and H) Spearman rank correlation analysis of CD44 expression with SUVmean (G) and SUVmax (H) of total tubers. CD44 expression was negatively correlated with SUVmean (correlation coefficient = −0.5124, *p* < 0.01) and SUVmax (correlation coefficient = −0.4779, *p* < 0.01) in total tubers. Epi, epileptogenic; SUVmax, maximum standardized uptake value; SUVmean, mean standardized uptake value.
**Figure S4:** SUVs were decreased in Epi tubers compared with non‐Epi tubers, and were negatively correlated with CD44 expression in cortical tubers. (A and B) Decreased SUVmean (A) and SUVmax (B) in resected Epi tubers (*n* = 37) compared with non‐Epi tubers (*n* = 20) from patients with tuberous sclerosis complex. Statistical analysis was conducted with two‐tailed unpaired Student's t‐test. Data are shown as means ± SEM. *p < 0.05. (C and D) Spearman rank correlation analysis of CD44 expression with the SUVmean (C) and SUVmax (D) of non‐Epi tubers. CD44 expression was negatively correlated with SUVmean (correlation coefficient = −0.5426, *p* < 0.05) and SUVmax (correlation coefficient = −0.5538, *p* < 0.05) in non‐Epi tubers. Epi, epileptogenic; SUV, standardized uptake value; SUVmax, maximum SUV; SUVmean, mean SUV.
**Figure S5:** CD44 expression in neocortical seizures model. (A) Western blot of CD44 in primary motor cortex of AAV‐Con mice. (B) Western blot analysis showed there were increased OD of CD44 in the primary motor cortex of neocortical seizures model than controls. *n* = 5 for each group. Statistical tests used for analysis were two‐tailed unpaired t‐tests. Data were shown as means ± SEM. *p < 0.05. OD, optical density.
**Figure S6:** The frequency of sEPSC and sIPSC in mice and human brain slices. (A) The sEPSC frequency was increased in both epileptic mice and human brain slices than non‐epileptic brain slices. (B) The sIPSC frequency was decreased in both epileptic mice and human brain slices than non‐epileptic brain slices. *n* = 14 cells of mice, *n* = 6 cells of non‐Epi tubers, and *n* = 10 cells of Epi tubers. Statistical tests used for analysis were two‐tailed unpaired *t*‐tests. Data were shown as means ± SEM. *p < 0.05, **p < 0.01. Epi, epileptogenic; EPSC, excitatory postsynaptic current; IPSC, inhibitory postsynaptic current.
**Figure S7:** CD44 expression in primary cortical neurons treated with KA and CD44 monoclonal antibody. (A) Western blot of CD44 in cultured primary cortical neurons treated with KA and CD44 monoclonal antibody. (B) Western blot analysis showed KA treatment increased OD of CD44, and CD44 monoclonal antibody rescued the increased CD44 induced by KA in primary cortical neurons. *n* = 6 for each group. Significance was calculated using ANOVA, followed by Tukey's test. Data were shown as means ± SEM. **p < 0.01. OD, optical density.
**Figure S8:** CASK expression was decreased in cultured cortical neurons treated with LV‐shCASK than LV‐NC. (A) Representative image of GFP‐positive cells in cultured cortical neurons transfected with LV‐NC and LV‐shCASK. Scale bar, 25 μm. (B) Western blot of CASK in cultured primary cortical neurons treated with LV‐shCASK and LV‐NC. (C) Western blot analysis showed there were decreased OD of CASK in primary cortical neurons treated with LV‐shCASK than LV‐NC. *n* = 6 for each group. Statistical tests used for analysis were two‐tailed unpaired *t*‐tests. Data were shown as means ± SEM. **p < 0.01. OD, optical density.
**Figure S9:** CD44–CASK pathway acted as a potential biomarker of human Epi tubers. (A–C) ROCs for identifying the Epi tubers from multiple cortical tubers based on CD44 (A), CASK (B) and combined CD44 and CASK (C) expression. The curve indicated that both CD44 and CASK could predict the Epi tubers (CD44: AUC = 0.82 ± 0.1011, *p* < 0.05; CASK: AUC = 0.78 ± 0.1058, *p* < 0.05), and combined CD44 and CASK performed better than CD44 and CASK alone (AUC = 0.90 ± 0.0805, *p* < 0.01). The optimal decision threshold of combined CD44 and CASK was 0.44. Epi, epileptogenic.
**Figure S10:** Correlation between mTORC1 activation and CD44–CASK upregulation in TSC tubers and epileptic mice. (A) Western blot of p‐S6 in resected Epi tubers and non‐Epi tubers from patients with TSC. (B) Western blot analysis showed that OD of p‐S6 was increased in Epi tubers compared with non‐Epi tubers. *n* = 10 for each group. (C) Western blot of p‐S6 in KA‐induced epileptic mouse. (D) Western blot analysis showed that OD of p‐S6 was increased in KA‐treated epileptic mice compared with controls. *n* = 6 for each group. (E) Spearman rank correlation analysis revealed a positive correlation between p‐S6 and CD44 expression (correlation coefficient = 0.7258, *p* < 0.05). (F) Spearman rank correlation analysis revealed a positive correlation between p‐S6 and CASK expression (correlation coefficient = 0.7930, *p* < 0.01). (G‐I) qRT‐PCR results showed upregulation of STAT3, HIF‐1α and NF‐κB in Epi tubers. *n* = 6 for each group. Data are shown as means ± SEM. *p < 0.05, **p < 0.01. Epi, epileptogenic; OD, optical density.
**Table S1:** The sequences of qRT‐PCR primers used in this study.
**Table S2:** Clinical features of patients with TSC.
**Table S3:** The differences between epileptogenic tubers and non‐epileptogenic tubers.
**Table S4:** Clinical features of patients with TLE and FLE.
**Table S5:** Clinical features of controls.
**Table S6:** Prediction of CD44–CASK pathway to the surgical outcomes in TSC.

## Data Availability

The RNA‐sequencing data of resected cortical tubers from TSC patients may be found in the National Centre for Biotechnology Information under accession number PRJNA1288162 or at the following URL: http://www.ncbi.nlm.nih.gov/sra/PRJNA1288162. The RNA‐sequencing data of the cultured mouse cortical neurons may be found in the National Centre for Biotechnology Information under accession number PRJNA1281274 or at the following URL: http://www.ncbi.nlm.nih.gov/sra/PRJNA1281274. Other data generated in this study are available upon reasonable request from the corresponding authors.
