# Supplementary material for: CD44 Predisposes the Epileptogenesis of Cortical Tubers by Regulating CASK and Acts as a Potential Biomarker to Identify Epileptogenic Tuber in Tuberous Sclerosis Complex
Source: CNS Neurosci Ther. 2026 Nov 9;32(11):e71262. doi: 10.1002/cns.71262 (PMC13748824; doi:10.1002/cns.71262)
Supplement: Supplementary file 1 [file CNS-32-e71262-s001.docx]

**Supplementary Figures**

**Figure S1.** Flowchart of patient inclusion and exclusion criteria. A total of 34 patients with tuberous sclerosis complex presented to Xinqiao hospital from June 2017 to January 2023, and 4 patients without intractable epilepsy, 4 patients without sufficient clinical information and 1 patient with imaging contraindications were excluded. In total, 25 patients were enrolled in the current study. Epi, epileptogenic.

**Figure S2.** **CD44 expression in patients with TLE and FLE, and controls.** **(A)** Western blot analysis of CD44 in TLE, FLE and controls. **(B)** Western blotting showed no significance difference in the optical density values of CD44 in TLE (n=5), FLE (n=4) or controls (n=4). TLE, temporal lobe epilepsy; FLE, frontal lobe epilepsy.

**Figure S3. Correlation analyses of CD44 expression and clinical features of Epi tubers. (A-C)** Spearman rank correlation analysis between CD44 expression (optical density of CD44) and size **(A)**, seizures frequency **(B)** and seizures duration **(C)** of Epi tubers. There was no correlation between CD44 expression and size of Epi tubers (correlation coefficient =0.1783, P=0.29), but CD44 expression was positively correlated with seizure frequency (correlation coefficient =0.4058, P<0.05) and seizures duration (correlation coefficient =0.3941, P<0.05). **(D)** Examples of preoperative localization of Epi and non-Epi tubers on imaging. **(E and F)** Spearman rank correlation analysis of CD44 expression with SUVmean **(E)** and SUVmax **(F)** of Epi tubers. CD44 expression was negatively correlated with SUVmean (correlation coefficient =-0.4345, P<0.01) and SUVmax (correlation coefficient =-0.3705, P<0.05) in Epi tubers. **(G and H)** Spearman rank correlation analysis of CD44 expression with SUVmean **(G)** and SUVmax **(H)** of total tubers. CD44 expression was negatively correlated with SUVmean (correlation coefficient =-0.5124, P<0.01) and SUVmax (correlation coefficient =-0.4779, P<0.01) in total tubers. Epi, epileptogenic; SUVmax, maximum standardized uptake value; SUVmean, mean standardized uptake value.

**Figure S4.** **SUVs were decreased in Epi tubers compared with non-Epi tubers, and were negatively correlated with CD44 expression in cortical tubers. (A and B)** Decreased SUVmean **(A)** and SUVmax **(B)** in resected Epi tubers (n=37) compared with non-Epi tubers (n=20) from patients with tuberous sclerosis complex. Statistical analysis was conducted with two-tailed unpaired Student’s t-test. Data are shown as means ± SEM. *P<0.05. **(C and D)** Spearman rank correlation analysis of CD44 expression with the SUVmean **(C)** and SUVmax **(D)** of non-Epi tubers. CD44 expression was negatively correlated with SUVmean (correlation coefficient =-0.5426, P<0.05) and SUVmax (correlation coefficient =-0.5538, P<0.05) in non-Epi tubers. Epi, epileptogenic; SUV, standardized uptake value; SUVmax, maximum SUV; SUVmean, mean SUV.

**Figure S5.** **CD44 expression in** **neocortical seizures model. (A)** Western blot of CD44 in primary motor cortex of AAV-Con mice. **(B)** Western blot analysis showed there were increased OD of CD44 in the primary motor cortex of neocortical seizures model than controls. n=5 for each group. Statistical tests used for analysis were two-tailed unpaired t-tests. Data were shown as means ± SEM. *P<0.05. OD, optical density.

**Figure S6.** **The frequency of sEPSC and sIPSC in mice and human brain slices.** **(A)** The sEPSC frequency was increased in both epileptic mice and human brain slices than non-epileptic brain slices. **(B)** The sIPSC frequency was decreased in both epileptic mice and human brain slices than non-epileptic brain slices. n=14 cells of mice, n=6 cells of non-Epi tubers, and n=10 cells of Epi tubers. Statistical tests used for analysis were two-tailed unpaired t-tests. Data were shown as means ± SEM. *P<0.05, **P<0.01. EPSC, excitatory postsynaptic current; IPSC, inhibitory postsynaptic current; Epi, epileptogenic.

**Figure S7. CD44 expression in primary cortical neurons treated with KA and CD44 monoclonal antibody.** **(A)** Western blot of CD44 in cultured primary cortical neurons treated with KA and CD44 monoclonal antibody. **(B)** Western blot analysis showed KA treatment increased OD of CD44, and CD44 monoclonal antibody rescued the increased CD44 induced by KA in primary cortical neurons. n=6 for each group. Significance was calculated using ANOVA, followed by Tukey’s test. Data were shown as means ± SEM. **P<0.01. OD, optical density.

**Figure S8.** **CASK expression was decreased in cultured cortical neurons treated with LV-shCASK than LV-NC.** **(A)** Representative image of GFP-positive cells in cultured cortical neurons transfected with LV-NC and LV-shCASK. Scale bar, 25 μm. **(B)** Western blot of CASK in cultured primary cortical neurons treated with LV-shCASK and LV-NC. **(C)** Western blot analysis showed there were decreased OD of CASK in primary cortical neurons treated with LV-shCASK than LV-NC. n=6 for each group. Statistical tests used for analysis were two-tailed unpaired t-tests. Data were shown as means ± SEM. **P<0.01. OD, optical density.

**Figure S9.** **CD44-CASK pathway acted as a potential biomarker of human Epi tubers.** **(A-C)** ROCs for identifying the Epi tubers from multiple cortical tubers based on CD44 **(A)**, CASK **(B)** and combined CD44 and CASK **(C)** expression. The curve indicated that both CD44 and CASK could predict the Epi tubers (CD44: AUC =0.82 ± 0.1011, P<0.05; CASK: AUC =0.78 ± 0.1058, P<0.05), and combined CD44 and CASK performed better than CD44 and CASK alone (AUC =0.90 ± 0.0805, P<0.01). The optimal decision threshold of combined CD44 and CASK was 0.44. Epi, epileptogenic.

**Figure S10. Correlation between mTORC1 activation and CD44-CASK upregulation in TSC tubers and epileptic mice. (A)** Western blot of p-S6 in resected Epi tubers and non-Epi tubers from patients with TSC. **(B)** Western blot analysis showed that OD of p-S6 was increased in Epi tubers compared with non-Epi tubers. n=10 for each group. **(C)** Western blot of p-S6 in KA-induced epileptic mouse. **(D)** Western blot analysis showed that OD of p-S6 was increased in KA-treated epileptic mice compared with controls. n=6 for each group. **(E)** Spearman rank correlation analysis revealed a positive correlation between p-S6 and CD44 expression (correlation coefficient =0.7258, P<0.05). **(F)** Spearman rank correlation analysis revealed a positive correlation between p-S6 and CASK expression (correlation coefficient =0.7930, P<0.01). **(G-I)** qRT-PCR results showed upregulation of STAT3, HIF-1α and NF-κB in Epi tubers. n=6 for each group. Data are shown as means ± SEM. *P<0.05, **P<0.01. OD, optical density; Epi, epileptogenic.

**Supplementary Tables**

| **Supplementary Table 1** The sequences of qRT-PCR primers used in this study. | | | |
| --- | --- | --- | --- |
| **Gene name** | | **Forward** | **Reverse** |
| Human | ANXA2 | GAGCGGGATGCTTTGAACATT | TAGGCGAAGGCAATATCCTGT |
|  | TNC | TCCCAGTGTTCGGTGGATCT | TTGATGCGATGTGTGAAGACA |
|  | CXCL10 | GTGGCATTCAAGGAGTACCTC | TGATGGCCTTCGATTCTGGATT |
|  | ITGB4 | CTCCACCGAGTCAGCCTTC | CGGGTAGTCCTGTGTCCTGTA |
|  | CD44 | CTGCCGCTTTGCAGGTGTA | CATTGTGGGCAAGGTGCTATT |
|  | PRP2 | GGACAGACACGTCAGCAAACT | GGGAGGGGCTTATATTGGCAT |
|  | NTRK1 | AACCTCACCATCGTGAAGAGT | TGAAGGAGAGATTCAGGCGAC |
|  | STAT3 | GTGAAAGCAGCAAAGAAGGAGG | CCGACAATACTTTCCGAATGCC |
|  | HIF-1α | TCATCAGTTGCCACTTCCACATA | ATCCAAATCACCAGCATCCAGAA |
|  | NF-κB | GAGGATGGGATCTGCACTGTAAC | GTGCACCAAGAGTCCAGGATTAT |
|  | GAPDH | TCAAGAAGGTGGTGAAGCAGG | AGCGTCAAAGGTGGAGGAGTG |
| Mouse | CASK | TAGCAGCAGAAAGAGCCGTC | TCCAGCAGTGTGTGAAGATGT |
|  | NRXN3 | AGCGTCCCTGTGAAAATGGT | CGTTCTCCTCTCGTGCTTGT |
|  | HOMER1 | CTCTCCACCTCCTCTCTGCT | ATGCTACTGCCTCCCTTACG |
|  | GRIN1 | TACCACCCTACTCCCACCAG | TTCTCTGCCTTGGACTCACG |
|  | CAMK2A | CCCCAAAGTCACAGAGCCAT | CAGGCAGCAGAGTAGTGGAC |
|  | LIN7C | AGTGAGTGTTGAAGGGGAGC | CTGTTGTCTGCGTTTTGCTGA |
|  | GAPDH | AAGAGGGATGCTGCCCTTAC | GTTCACACCGACCTTCACCA |

| **Supplementary Table 2** Clinical features of patients with TSC. | | | | | | | | | | | |  |
| --- | --- | --- | --- | --- | --- | --- | --- | --- | --- | --- | --- | --- |
| **Case** | **Gender** | **Age at surgery**  **(year)** | **Seizure frequency (month)** | **Epilepsy duration (s)** | **Numbers of Epi tubers** | **Numbers of Non-epi tubers** | **Seizure (type)** | **Localization of epi-tubers** | **Engel's (class)** | **Application in this study** |  |  |
| 1 | F | 6 | 15 | 124 | 3 | 2 | PS; Tonic | Frontal lobe; Parietal lobe | II | WB, IHC, |  |  |
| 2 | M | 2 | 5 | 53 | 2 | 4 | PS; IS | Frontal lobe | II | WB, IHC, RNA-seq, qPCR |  |  |
| 3 | M | 5 | 10 | 80 | 1 | 2 | PS | Occipital lobe | I | WB, IHC, RNA-seq |  |  |
| 4 | M | 4 | 42 | 150 | 3 | 3 | PS; GTCS | Frontal lobe; Parietal lobe | I | WB, IHC, |  |  |
| 5 | F | 18 | 6.67 | 14 | 1 | 2 | GTCS | Temporal lobe | I | WB, IHC, RNA-seq, qPCR |  |  |
| 6 | F | 1 | 30 | 4 | 1 | 3 | GTCS; IS | Frontal lobe | II | WB, IHC, RNA-seq, qPCR |  |  |
| 7 | M | 7 | 3 | 8 | 1 | 0 | PS | Occipital lobe | I | WB, IHC, |  |  |
| 8 | F | 21 | 12.85 | 15 | 1 | 2 | PS | Frontal lobe | I | WB, IHC, RNA-seq |  |  |
| 9 | F | 1.5 | 130 | 21 | 1 | 0 | PS; IS | Occipital lobe | I | WB, IHC, RNA-seq |  |  |
| 10 | F | 0.5 | 5.625 | 124 | 1 | 2 | PS; IS | Occipital lobe | II | WB, IHC, |  |  |
| 11 | M | 13 | 97 | 9 | 1 | 1 | GTCS | Temporal lobe | I | WB, IHC, RNA-seq, qPCR |  |  |
| 12 | F | 7 | 75 | 13 | 2 | 3 | GTCS | Frontal lobe; Parietal lobe | I | WB, IHC, RNA-seq |  |  |
| 13 | M | 16 | 50 | 29 | 1 | 0 | PS | Occipital lobe | I | WB, IHC, RNA-seq, qPCR |  |  |
| 14 | F | 9 | 112.5 | 57 | 1 | 2 | PS | Parietal lobe | I | WB, IHC, |  |  |
| 15 | F | 3 | 80 | 193 | 2 | 1 | PS; Tonic | Frontal lobe; Parietal lobe | II | WB, IHC, RNA-seq |  |  |
| 16 | M | 13 | 7.5 | 16 | 1 | 3 | PS | Occipital lobe | I | WB, IHC, RNA-seq, qPCR |  |  |
| 17 | M | 17 | 120 | 36 | 1 | 1 | PS | Parietal lobe | II | WB, IHC, |  |  |
| 18 | F | 26 | 67.5 | 97 | 1 | 2 | GTCS | Parietal lobe | I | WB, IHC, RNA-seq |  |  |
| 19 | F | 6 | 72 | 24 | 2 | 1 | PS | Frontal lobe | I | WB, IHC, |  |  |
| 20 | M | 3 | 12 | 61 | 2 | 4 | PS; IS | Occipital lobe | I | WB, IHC, |  |  |
| 21 | M | 4 | 9 | 76 | 1 | 0 | GTCS | Frontal lobe | I | WB, IHC, |  |  |
| 22 | M | 11 | 112 | 12 | 3 | 2 | PS | Frontal lobe | II | WB, IHC, |  |  |
| 23 | F | 13 | 27 | 40 | 1 | 2 | PS; Tonic | Parietal lobe | I | WB, IHC, |  |  |
| 24 | M | 7 | 42 | 31 | 2 | 3 | PS | Occipital lobe | I | WB, IHC, |  |  |
| 25 | F | 5 | 16 | 65 | 1 | 1 | GTCS | Frontal lobe | I | WB, IHC, |  |  |
| Abbreviations: F, Female; M, Male; PS, partial seizure; Tonic, tonic seizure; IS, infantile spasm; GTCS, generalized tonic-clonic seizure; WB, Western blot; IHC,immunohistochemistry; RNA-seq,high-throughput RNA-sequence; qPCR,quantitative real-time polymerase chain reaction | | | | | | | | | | | |  |
|  |  |  |  |  |  |  |  |  |  |  |  |  |

| **Supplementary Table 3** The differences between Epileptogenic tubers and Non-epileptogenic tubers. | | | | | | | | | | | | |  |
| --- | --- | --- | --- | --- | --- | --- | --- | --- | --- | --- | --- | --- | --- |
| **Clinical characteristics** | | | | | **Epileptogenic tubers (n=37)** | | | | **Non-epileptogenic tubers (n=20)** | | | |  |
| Size (>3cm) | | | | | 30(81.1) | | | | 9(45.0) | | | |  |
| Calcification | | | | | 25(67.6) | | | | 11(55.0) | | | |  |
| Cyst-like | | | | | 21(56.8) | | | | 6(30.0) | | | |  |
| Epileptic discharge | | | | | 37(100) | | | | 0(0) | | | |  |
| Seizure frequency (per month) | | | | | 3-130 | | | | — | | | |  |
| Seizure duration (s) | | | | | 4-193 | | | | — | | | |  |
| Note: The size, calcification and cyst-like properties of tubers were evaluated on MRI images, and the epileptic discharges are recoded by stereoelectroencephalogram (SEEG) recordings. Values are n (%). | | | | | | | | | | | | |  |
| **Supplementary Table 4** Clinical features of patients with TLE and FLE. | | | | | | | | | | | |  |  |
| **Case** | **Gender** | **Diagnosis** | **Age at surgery**  **(year)** | **Seizure frequency (month)** | | **Epilepsy duration (s)** | **Seizure (type)** | **Localization of surgical resection** | | **Engel's (class)** | **Application in this study** |  |  |
| 1 | M | TLE | 42 | 32 | | 51 | PS; Tonic | Temporal lobe | | I | WB |  |  |
| 2 | F | TLE | 29 | 43 | | 24 | PS | Temporal lobe | | I | WB |  |  |
| 3 | M | TLE | 51 | 13 | | 36 | PS | Temporal lobe | | I | WB |  |  |
| 4 | M | TLE | 23 | 29 | | 97 | PS; GTCS | Temporal lobe | | II | WB |  |  |
| 5 | F | TLE | 31 | 42 | | 64 | GTCS | Temporal lobe | | I | WB |  |  |
| 6 | M | FLE | 19 | 38 | | 71 | GTCS | Frontal lobe | | I | WB |  |  |
| 7 | F | FLE | 32 | 12 | | 39 | PS | Frontal lobe | | I | WB |  |  |
| 8 | F | FLE | 26 | 25 | | 68 | GTCS | Frontal lobe | | I | WB |  |  |
| 9 | M | FLE | 35 | 42 | | 29 | PS; Tonic | Frontal lobe | | II | WB |  |  |
| Abbreviations: F, Female; M, Male; PS, partial seizure; Tonic, tonic seizure; GTCS, generalized tonic-clonic seizure; WB, Western blot. | | | | | | | | | | | |  |  |
|  |  |  |  |  |  |  |  |  |  |  |  |  | |

| **Supplementary Table 5** Clinical features of Controls. | | | | | |  |
| --- | --- | --- | --- | --- | --- | --- |
| **Case** | **Gender** | **Age（year）** | **Cause of death** | **Localization of resection** | **Application in this study** |  |
| 1 | F | 45 | Motor vehicle accident | Frontal lobe | WB |  |
| 2 | M | 37 | Motor vehicle accident | Temporal lobe | WB |  |
| 3 | M | 51 | Motor vehicle accident | Frontal lobe | WB |  |
| 4 | M | 21 | Motor vehicle accident | Temporal lobe | WB |  |
| Abbreviations: F, Female; M, Male | | | | | |  |
|  |  |  |  |  |  |  |

| **Supplementary Table 6** Prediction of CD44-CASK pathway to the surgical outcomes in TSC. | | | | | | | | | |
| --- | --- | --- | --- | --- | --- | --- | --- | --- | --- |
| **Biomarkers** | **1-year follow-up (n=7)** | | | **5-year follow-up (n=7)** | | | **8-year follow-up (n=7)** | | |
|  | CD44 | CASK | Combined CD44 and CASK | CD44 | CASK | Combined CD44 and CASK | CD44 | CASK | Combined CD44 and CASK |
| **SF** | **7** | | | **6** | | | **5** | | |
| **No SF** | **0** | | | **1** | | | **2** | | |
| **AUC** | **—** | | | **0.68** | **0.72** | **0.78** | **0.75** | **0.81** | **0.86** |
